# Genetic Susceptibility Within the Immune System Beyond Conventional Risk Factors: An Integrative Analysis in a Kazakh Cohort

**DOI:** 10.3390/biomedicines14102387

**Published:** 2026-10-19

**Authors:** Aygerim Serikzhan, Zhannur Abilova, Ainur Akhmetova, Aidana Gabdulkayum, Asset Z. Daniyarov, Saule E. Rakhimova, Ulan Kozhamkulov, Ulykbek Kairov, Ainur Akilzhanova, Dauren Yerezhepov

**Affiliations:** 1 Center for Life Sciences, National Laboratory Astana, Nazarbayev University, Astana 010000, Kazakhstan; 2 Department of General Biology and Genomics, Institute of Natural Sciences, L.N. Gumilyov Eurasian National University, Astana 010000, Kazakhstan

**Keywords:** genetic association study, Kazakh population, tuberculosis, immunogenetic array, polygenic risk score

## Abstract

**Background/Objectives**: Genetic factors are known to influence overall immune vulnerability to diseases, such as tuberculosis (TB); however, isolating the genetic component can be challenging as social and environmental causes, such as smoking, alcohol consumption, and unemployment, also drive disease risk. To reduce heterogeneity related to these factors, we performed a targeted immunogenetic association study of TB susceptibility in a Kazakh case–control cohort selected among individuals without the typical socioeconomic and nutritional risk factors linked to TB. **Methods**: We performed genotyping using an immune-focused targeted array; after quality control at sample and variant level, removal of ancestry-based outliers, and adjustment for population stratification, the final dataset included 164 participants (84 cases, 80 controls) and 181,595 variants. We performed four complementary analyses: single-variant association testing using logistic regression with Firth correction where required adjusted for sex and genetic ancestry, gene-based aggregation testing, functional annotation, and polygenic risk scoring using existing multi-ancestry TB genetic resources. **Results**: While no variant reached formal significance, the top nominal signal, rs1560036 (OR = 2.98, *p* = 2.4 × 10^−5^), lies within CPLX2, which ranked among the top genes in gene-based analysis, a result driven largely by the same variant. A nominally associated variant in the classical MHC region, rs9263600, is separately reported in GTEx as a cis-eQTL for HLA-B and HLA-C. Similarly, polygenic risk scores showed directionally consistent but statistically non-significant associations with case status (Asia-base AUC = 0.560; Europe-base AUC = 0.537). **Conclusions**: Although our study was not adequately powered to detect small-to-moderate genetic effects, it offers an important perspective on TB genetics in a population underrepresented in existing research. These findings provide an openly available dataset and candidate signals for replication in larger, independent cohorts.

## 1. Introduction

The tuberculosis (TB) infection rate is a notable challenge for the medical systems of Central Asia, including Kazakhstan. The number of new cases in Kazakhstan fell from 168 per 100,000 residents in the year 2000 to 45 per 100,000 residents in 2024 [1]; but this frequency is still higher than the limit of 10 per 100,000 residents that the World Health Organization uses to identify a low TB-burden environment [2].

Host genetic factors are well known to influence variation in TB susceptibility beyond conventional epidemiological and clinical risk factors: association studies have implicated immune-pathway loci, most notably the human leukocyte antigen (HLA) region [3], and twin and family studies have established a heritable component to TB risk [4]. The largest effort to date, the International Tuberculosis Host Genetics Consortium multi-ancestry meta-analysis of 14,153 cases and 19,536 controls across African, European, and Asian cohorts, identified a substantial, ancestry-shared polygenic contribution to TB risk [3]. However, host genetic studies of TB susceptibility in Central Asian populations remain limited. In Kazakhstan, previous studies have used candidate-gene designs, genotyping small numbers of pre-selected polymorphisms by real-time PCR. These include variants in VDR, TLR8 and MARCO [5]; VDR polymorphisms in Kazakhs from Almaty and the Almaty region [6]; variants across the vitamin D pathway [7]; VDR and TLR polymorphisms in relation to vitamin D status [8]; and IFNG, VDR and TLR8 polymorphisms in a cohort that included household contacts [9]. A candidate-gene study of NRAMP1, VDR and HLA class II genes has also been reported in ethnic Kazakhs in China [10]. In our literature search via Google Scholar, we identified no genome-wide or array-based association study of TB susceptibility in a Kazakh population, and Kazakh cohorts are not represented in the base GWAS used to construct polygenic risk scores (PRSs) for this trait [3].

This gap has practical consequences, because genetic association findings, and PRSs built from them, often transfer poorly across ancestry groups owing to differences in linkage disequilibrium (LD) structure, allele frequencies, and causal-variant architecture [11]. The Kazakh population carries patterns of biomedically relevant variation distinct from both East Asian and European populations [12], so the LD and allele-frequency assumptions of existing TB GWAS cannot be taken to hold in this population. We addressed this directly in the study design: LD for gene-based testing was estimated from a Kazakh whole-genome reference panel rather than a public panel, the cohort’s ancestry was assessed against an independent Kazakh reference population, and PRSs were derived separately from Asian- and European-ancestry base GWAS to assess whether either is detectable in this cohort.

In this study, we performed an exploratory case–control association study of pulmonary TB susceptibility in a Kazakh cohort restricted to participants without conventional TB risk factors (Section 2.1) to reduce heterogeneity related to selected environmental and clinical risk factors. Genotyping used an immune-pathway-focused array (Infinium ImmunoArray-24v2) rather than a genome-wide array. This design concentrates coverage on immune and inflammatory genes, including the MHC region, where TB associations have been most consistently reported [3], at the cost of non-uniform coverage elsewhere in the genome, which limits gene-based testing outside immune regions and reduces the overlap between target and base-GWAS variants available for PRS construction. Within these constraints, we report single-variant and LD-clumped association results, gene-based tests, functional annotation of suggestive loci, and the performance of published PRSs in this cohort. Given the modest sample size, we frame this study as hypothesis-generating and report post hoc statistical power alongside the results to support interpretation of the null findings.

## 2. Materials and Methods

### 2.1. Study Population

We conducted a case–control study. Cases were patients with pulmonary TB recruited at local TB dispensaries and diagnosed according to national clinical guidelines on the basis of clinical presentation and chest radiography, with or without bacteriological confirmation by sputum microscopy. The case group included both new and recurrent episodes, and the same case definition was applied to all cases. To reduce heterogeneity related to known risk factors, we included only participants without major risk factors as defined by the CDC and WHO, i.e., meeting all of the inclusion criteria and none of the exclusion criteria listed below. Specifically, participants were required to be aged between 18 and 65 years, have a stable income, be employed (excluding harmful and high-grade physical activity work types and prison workers), and have a normal BMI (18.5–25.0) before disease onset, based on self-reported usual weight. Exclusion criteria included malnutrition, any type of chronic upper respiratory tract disease (bronchitis, obstructive pulmonary disease), immunodeficiency-associated diseases, hepatitis virus infection, immune disorder, diabetes mellitus, smoking, drug abuse, contact with TB, HIV infection, alcohol overconsumption, maternity leave, being a prisoner in the past 3 years, and migration in the past 3 years. These criteria were applied through eligibility restriction at recruitment rather than through post hoc statistical adjustment, given that each additional covariate reduces the power available to detect a genetic effect at this sample size (N = 164). In addition, if factors such as income or employment influenced which individuals were identified as cases, statistical adjustment after enrollment would not fully remove the resulting bias. Restriction was intended to reduce heterogeneity related to these factors; it does not eliminate confounding, and its implications for selection bias and generalizability are discussed in Section 4.

Controls had no family history of pulmonary TB in first-degree relatives, no history of TB or registered TB contact in the Unified National Electronic Healthcare System (UNEHS) [13], a chest X-ray without abnormalities within 3 months before recruitment, and a BCG scar. Because of resource constraints, controls were not tested for latent TB infection; a normal chest X-ray excludes radiologically apparent disease but not latent infection. Each control was individually matched to a case by sex, ethnicity and region of residence, and by age within ±2 years. The same inclusion and exclusion criteria were applied to cases and controls.

All subjects were unrelated Kazakhs with available clinical data. Eligibility was assessed for cases and controls using the same criteria and the same procedures: a structured questionnaire administered by trained staff during an in-person interview, anthropometric measurements taken at the same visit, and review of medical records. “Good social and epidemiological status” denotes meeting all the inclusion criteria and none of the exclusion criteria listed above. Stable income was defined as permanent employment for more than one year with self-reported sufficient income. Harmful and high-intensity physical work was defined as self-reported current or previous employment in industrial production, mining, construction or demolition, or penitentiary institutions. Alcohol overconsumption was defined as heavy episodic drinking, i.e., consumption of ≥60 g of pure alcohol on at least one occasion in the past 30 days [14]. Smoking and drug use were assessed by self-report. Contact with TB was defined as household or other close contact with a person with active TB at any time, ascertained from the participant’s records in the Unified National Electronic Healthcare System (UNEHS) and the National Registry of Tuberculosis Patients [13], supplemented by self-report at interview.

The study was conducted in accordance with the Declaration of Helsinki and approved by the Local Ethics Committee of the Private Institution “National Laboratory Astana” (Protocol N04-2020, approved 26 August 2020; Protocol N03-2024, approved 2 October 2024). All subjects signed a written informed consent form.

A total of 192 individuals were genotyped. Following genotyping quality control and ancestry-based outlier removal (Section 2.4), a final analytic sample of 164 individuals (84 cases, 80 controls; 92 males, 72 females) and 181,595 SNPs was retained for association analysis (Table 1, Section 3.1).

### 2.2. DNA Isolation

Blood was collected into tubes containing K2EDTA. DNA was extracted using the QIAamp DNA Mini extraction Kit (Qiagen GmbH, Hilden, Germany), according to the manufacturer’s instructions for DNA purification from whole blood, and stored at −20 °C. The quantity and quality of the DNA were evaluated using the NanoDrop 2000 UV spectrophotometer (ThermoFisher Scientific, Waltham, MA, USA). The exact quantity was measured by the Qubit BR Assay Kit (ThermoFisher Scientific, Waltham, MA, USA) on a Qubit v2.0 fluorometer. DNA integrity was tested on 1% agarose gel electrophoresis. DNA was stored at −20 °C in the Laboratory Biobank.

### 2.3. Genotyping

Genotyping was performed using the Illumina InfiniumImmunoArray-24v2 BeadChip (Illumina Inc., San Diego, CA, USA), an array enriched for single-nucleotide polymorphisms (SNPs) in immune and inflammatory pathway genes. Raw intensity data were processed and genotypes were called using Illumina’s array-analysis-cli software, with variants annotated against the GRCh38 human reference genome assembly.

### 2.4. Quality Control

The 192 raw per-sample genotype calls (247,814 SNPs) were merged into a multi-sample VCF, split at multiallelic sites and dbSNP-annotated (248,349 SNPs), after which variants with unresolved or suspect rsID mappings and duplicate identifiers were removed (248,168 and 248,162 SNPs, respectively), and ambiguous-strand (A/T, C/G) SNPs were excluded, yielding 220,595 SNPs across all 192 samples for PLINK-based quality control. Sample- and variant-level filtering was then applied in sequence: a per-variant call-rate filter removing variants genotyped in fewer than 95% of samples (PLINK –geno 0.05), followed by a per-sample call-rate filter removing samples genotyped at fewer than 95% of variants (PLINK –mind 0.05), together reducing the dataset to 185 samples and 217,647 variants; removal of samples with outlying heterozygosity, defined as more than 3 standard deviations from the sample-wide mean heterozygosity rate (mean = 0.2824, SD = 0.0059), which excluded 1 sample (184 samples remaining); removal of related individuals using the KING robust kinship estimator at a second-degree-or-closer relatedness cutoff (–king-cutoff 0.177) [15], leaving 179 samples; exclusion of variants showing differential missingness between cases and controls (PLINK –test-missing, *p* < 1 × 10^−5^), a check for genotyping artifacts rather than true association; exclusion of variants deviating from Hardy–Weinberg equilibrium, assessed in controls only (–hwe 1 × 10^−6^), leaving 179 samples and 217,646 variants; and exclusion of variants with minor allele frequency below 1% (–maf 0.01), yielding 179 samples and 181,595 variants entering ancestry PCA. Genetic sex was inferred from X-chromosome inbreeding coefficients (PLINK –impute-sex; F < 0.31 female, F > 0.43 male) and matched recorded sex in 161 of 162 individuals with available records; genetically inferred sex was used as the covariate for all individuals, and the lead association was unchanged without sex adjustment (Section 3.3).

Ancestry-informative SNPs for all subsequent PCA rounds (Section 2.5) were selected by linkage-disequilibrium pruning (PLINK –indep-pairwise 50 5 0.2: 50-SNP windows, 5-SNP step size, r^2^ < 0.2). The full sample and variant flow is summarized in Table 2, Section 3.1. All genotype processing and quality control used bcftools 1.24 [16] and PLINK v1.90b7.2 and PLINK2 [17].

### 2.5. Population Structure and Association Analysis

Two PCAs were performed, each on LD-pruned autosomal SNPs (PLINK –indep-pairwise 50 5 0.2); long-range LD regions were not excluded. First, an internal PCA within the study sample was used to identify and remove ancestry outliers; this was applied iteratively over three rounds, reducing the sample from 179 to 173, 168, and finally 164 individuals (Table 2). At each of three rounds, PCA was recomputed on the remaining samples, and individuals lying more than 4 standard deviations from the sample mean on PC1 or PC2 were removed. Outlier status was determined from the principal component scores alone, without reference to case/control status. Second, a joint PCA was performed on the final analytic set (*n* = 164) together with a public reference panel, to visualize the study sample’s genetic position relative to other populations rather than for outlier removal. This reference panel comprised 19 populations (Altaian, Azeri, Buryat, French, German, Han Chinese, Hazara, Koryak, Lezgin, Pathan, Polish, Russian Central, Spanish, Tatar, Turkmen, Tuvan, Ukrainian, Uygur, and Yakut) selected from the 163-population dataset compiled by Hellenthal et al. [18] and Busby et al. [19] and made available via Mendeley Data [20], merged with an additional Kazakh reference population from Serikzhan et al. [12].

The primary association model was logistic regression of case/control status on the additive allele count of hard-called array genotypes (no imputation), adjusted for genetically inferred sex and the first three ancestry principal components, performed in PLINK v2.00 (–glm firth-fallback). With this option, standard logistic regression is fitted for each variant, and Firth-penalized regression is used only when the standard model fails to converge; the lead variant, rs1560036, was analyzed with standard logistic regression, and applying Firth regression to all variants gave a similar estimate (Section 3.3). Individuals with a missing genotype at a given variant were excluded from that variant’s test. Because some matched pairs were incomplete after quality control, an unconditional model was used [21]; sex was included as a covariate and ethnicity was uniform, whereas age and region of residence were not included (see the section on limitations).

Three PCs were retained to correct for broad ancestry structure while limiting the loss of power from additional covariates at n = 164. The first three principal components explained 30.4% of the variance among the ten computed, and the eigenvalue spectrum declined gradually without a distinct elbow after outlier removal. In sensitivity analyses adjusting for PC1 alone or for PC1–PC10, the lead variant rs1560036 remained consistent in direction and significance (PC1 only: OR = 2.97, *p* = 2.49 × 10^−5^; PC1–PC3: OR = 2.98, *p* = 2.42 × 10^−5^; PC1–PC10: OR = 3.36, *p* = 1.31 × 10^−5^).

Age and BMI were not included in the primary model. Age was a matching variable and did not differ between cases and controls (Table 1). BMI was measured at enrollment, after disease onset, and is lower in active TB because of disease-related weight loss; adjusting for a variable influenced by the outcome may bias, rather than reduce, confounding of the genetic association.

Sensitivity analyses (Section 3.3) comprised the primary model without sex adjustment, the primary model additionally adjusted for age and for age plus BMI, Firth regression applied to all variants, a genotypic (additive plus dominance-deviation) model, and permutation-based empirical significance (PLINK v1.9). All used the same 164 individuals and variants, except that the permutation analyses were not covariate-adjusted and the age- and BMI-adjusted models included the 162 individuals with complete data.

Permutation analyses were performed in PLINK v1.9 by randomly permuting case/control labels across all 164 individuals, without stratification by covariates or matching variables and without covariate adjustment. Two procedures were used. First, adaptive permutation of the allelic chi-square test (–assoc –perm; up to 1,000,000 permutations) was used to obtain a point-wise empirical p-value (EMP1), calculated as (R + 1)/(N + 1), where R is the number of permutations with a test statistic at least as large as the observed one and N is the number of permutations. Second, 10,000 fixed max(T) permutations of the best-fitting test from PLINK’s –model procedure (genotypic, allelic, trend, dominant or recessive; –model –mperm 10,000) were used to obtain a point-wise EMP1 and a panel-wide corrected EMP2. EMP2 is the proportion of permutations in which the maximum test statistic across all variants tested was at least as large as the observed statistic for the variant, and therefore corrects for testing all variants on the panel.

### 2.6. Multiple Testing Correction and Statistical Power

Because standard genome-wide significance (*p* = 5 × 10^−8^) assumes approximately one million independent tests and is not calibrated for this 181,595-SNP targeted immune panel, an effective-number-of-tests correction was computed for the 180,388 autosomal SNPs using the simpleM method [22] (sliding window of 2000 SNPs, 500-SNP overlap, 99.5% cumulative variance threshold), yielding an effective number of independent tests (M_eff) of 16,319.25 and a panel-appropriate significance threshold of *p* < 3.06 × 10^−6^. Chromosome X SNPs (n = 1207) were analyzed separately because hemizygosity in males alters both genotype coding and the LD structure on which simpleM relies. Association testing on chromosome X used PLINK 2.0 default coding (males and females on a 0–2 scale, with males coded as hemizygous) with sex as a covariate; pseudo-autosomal regions were not analyzed separately. Because simpleM could not be applied to the mixed-ploidy data, we applied a Bonferroni correction to the number of LD-pruned chromosome X SNPs (568; r^2^ < 0.2), giving a threshold of 8.80 × 10^−5^. This threshold was defined after the autosomal analysis rather than prespecified. Applied jointly with the autosomal tests, the overall threshold would be 0.05/(16,319 + 568) = 2.96 × 10^−6^.

Post hoc statistical power was estimated using genpwr [23] across a range of plausible odds ratios and minor allele frequencies at both the panel-corrected and genome-wide significance thresholds, and, as a less stringent scenario, at 1 × 10^−5^ using the final case/control counts (84/80), to establish what effect sizes this sample size was realistically able to detect.

### 2.7. Independent Loci and Gene-Based Association Analysis

Because several SNPs reaching nominal significance in the primary model were expected to be correlated proxies of the same underlying signal rather than independent findings, LD clumping was performed in PLINK v1.9 (–clump; index threshold *p* < 5 × 10^−4^, r^2^ ≥ 0.1, 250 kb window) on the additive-term results of the primary model, with LD estimated from the 164 study participants using individuals with non-missing genotypes for each pair of variants.

Gene-based association testing was performed with MAGMA v1.10 [24] using the SNP-wise mean model on the p-values of the primary association model (N = 164). SNPs were mapped to genes with NCBI Gene definitions for GRCh38, using two annotation windows (10 kb upstream/10 kb downstream and 35 kb upstream/10 kb downstream). LD was estimated from an in-house Kazakh whole-genome-sequencing reference panel of 221 unrelated individuals, restricted to variants present on the array and filtered for minor allele frequency ≥1% and genotype missingness ≤5%. Array SNPs absent from the reference panel were excluded, so the analysis was restricted to the 170,090 variants (93.7%) represented in the reference. Chromosome X genes (n = 125) were included, with LD estimated from the reference genotypes without sex-specific modeling. Significance was assessed by Bonferroni correction for the number of genes tested (12,980 genes for the 10 kb/10 kb window and 15,015 for the 35 kb/10 kb window; thresholds 3.85 × 10^−6^ and 3.33 × 10^−6^, respectively).

### 2.8. Functional Annotation

The 39 SNPs nominally associated with TB status in the primary model (*p* < 5 × 10^−4^; Section 2.7) were functionally annotated using the Ensembl Variant Effect Predictor (VEP) [25] against the GRCh38 assembly, including nearest-gene and consequence annotation, population allele frequencies from gnomAD [26], and cross-referencing against previously reported trait associations in the NHGRI-EBI GWAS Catalog [27]. To assess potential gene-regulatory effects, each variant was further queried against the GTEx v8 (GRCh38) cis-eQTL dataset [28] using a custom Python (version 3.12) script: for each rsID, the corresponding GTEx variant identifier was retrieved via the GTEx Portal API (/dataset/variant endpoint), and this identifier was used to query all precomputed significant single-tissue cis-eQTL associations (/association/singleTissueEqtl endpoint), recording the associated gene(s), tissue(s), and eQTL *p*-value(s) for each variant.

### 2.9. Polygenic Risk Score

Because the base-GWAS summary statistics are reported on GRCh37 and the target genotype data on GRCh38, target data were lifted to GRCh37 using the UCSC liftOver tool; variants that failed to lift were recovered via the Ensembl GRCh37 REST API, restricting to primary chromosomes. Because SNP identifiers were inconsistent between target and base data (a mix of rsIDs and position-based IDs), variants were matched by chromosome and GRCh37 position. Allele identity was then verified during scoring: PLINK v1.9 (–score) used a variant only if the base effect allele matched one of the two target alleles at that position, so variants with mismatched alleles (e.g., strand flips) were excluded. Strand-ambiguous (A/T, C/G) variants had already been removed during quality control (Section 2.4).

Polygenic risk scores were constructed separately from Asian-ancestry (5 cohorts) and European-ancestry (3 cohorts) fixed-effects meta-analysis summary statistics from the International Tuberculosis Host Genetics Consortium, generated using GWAMA [3,29]. Clumping-and-thresholding was applied to each base separately (PLINK –clump, p1 = p2 = 0.05, r^2^ ≥ 0.1, 250 kb), with LD estimated in the 164 study participants: of the matched variants, 8488 (Asia) and 10,312 (Europe) reached *p* < 0.05, yielding 3602 and 3850 independent SNPs, respectively (Supplementary Table S4). A single threshold (*p* < 0.05) was used, as is common for clumping-and-thresholding scores; given the small target sample, it was not optimized to avoid overfitting. Variant weights were the natural logarithm of the base-GWAS odds ratio for the effect allele, missing genotypes were replaced by their expected value based on the sample allele frequency (PLINK default), and scores were standardized to mean 0 and SD 1 within the cohort. Scores were evaluated by logistic regression and area under the receiver operating characteristic curve (AUC), both unadjusted and adjusted for PC1–PC3.

## 3. Results

### 3.1. Study Population and Quality Control

Of 192 genotyped individuals, 164 (84 cases, 80 controls; 92 males, 72 females) passed all quality-control and ancestry-outlier-removal steps and were retained for the primary analysis. Their characteristics are summarized in Table 1.

Cases and controls were similar in age (mean 30.6 ± 11.4 vs. 32.8 ± 11.3 years), sex distribution (56.0% vs. 56.3% male) and place of residence (52.4% vs. 46.3% urban), which is consistent with the matching design. Height did not differ between groups, but cases had a slightly lower body weight (59.6 ± 11.1 vs. 63.5 ± 10.1 kg) and BMI (21.0 ± 3.9 vs. 22.3 ± 2.8 kg/m^2^) than controls, as expected given weight loss associated with active TB. Residence was unknown for two controls.

**Figure 1:**
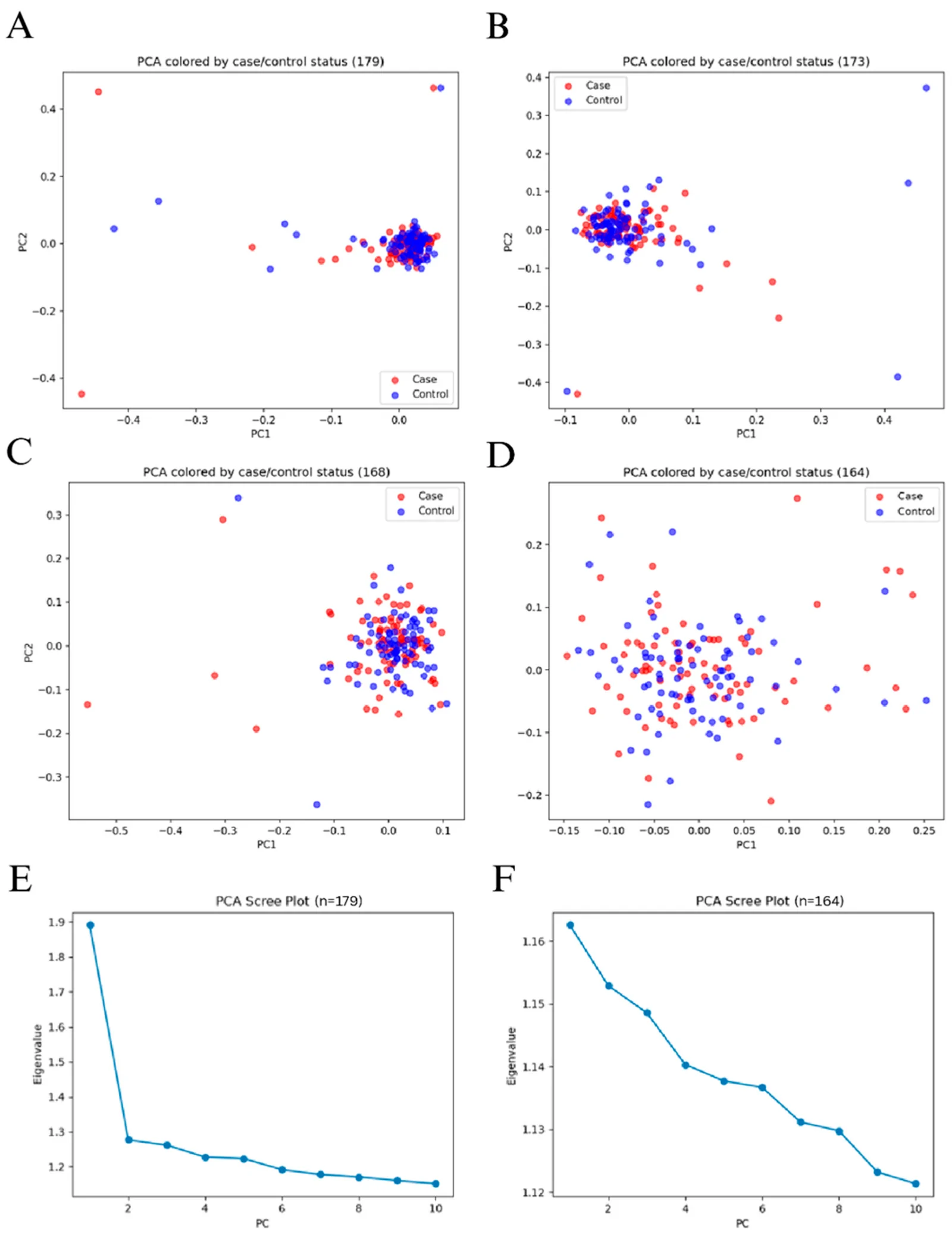
Population structure before and after iterative ancestry-outlier removal. (**A**) PC1 vs. PC2 before outlier removal (n = 179). (**B**) PC1 vs. PC2 after round 1 (n = 173). (**C**) PC1 vs. PC2 after round 2 (n = 168). (**D**) PC1 vs. PC2 after round 3, final analytic set (n = 164). (**E**) Scree plot before outlier removal. (**F**) Scree plot after outlier removal.

The effect of iterative outlier-removal procedure on the observed population structure is illustrated in Figure 1. Before outlier removal (Figure 1A; n = 179), a small number of individuals among both cases and controls fell far outside the main cluster along PC1 and PC2, consistent with the disproportionately large first eigenvalue seen in the corresponding scree plot (Figure 1E). Removing these individuals resolved the most extreme deviations, but two further rounds (Figure 1B, n = 173; Figure 1C, n = 168) were needed to progressively reveal and resolve smaller sets of residual outliers. After a third round (Figure 1D; final analytic set, n = 164), the sample formed a single, comparatively homogeneous cluster with no pronounced outliers along PC1 or PC2, and cases and controls remained well intermixed throughout. Across the three rounds, 15 individuals were removed (round 1: four cases, two controls; round 2: one case, four controls; round 3: two cases, two controls; six males and nine females in total). Removal was not associated with case/control status (Fisher’s exact test, *p* = 0.79) or sex (*p* = 0.28), and the final sample remained balanced (84 cases, 80 controls). This progressive homogenization was mirrored in the scree plots: eigenvalues after outlier removal (Figure 1F) were both substantially smaller in magnitude and declined more gradually across the first ten PCs than before removal (Figure 1E), consistent with the removal of a small number of individuals who had been disproportionately driving the early principal components.

### 3.2. Population Structure

**Figure 2:**
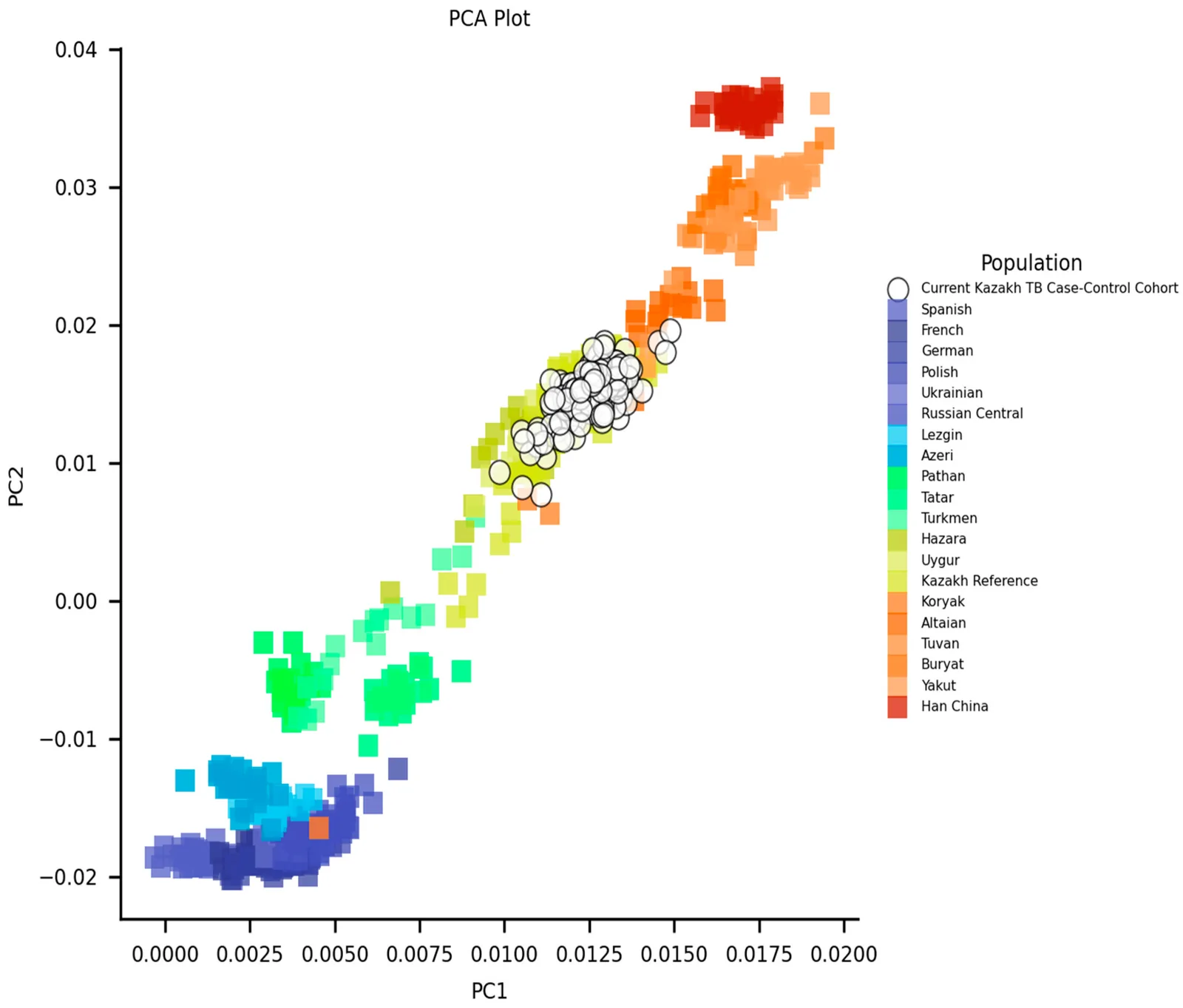
Joint principal component analysis (PCA) of the current Kazakh TB case–control cohort (n = 164) with public reference populations.

Final analytic cohort in the context of genetic variation across Eurasia is demonstrated in Figure 2. The reference populations formed a broad West-to-East genetic gradient. At one end sat European populations (Spanish, French, German, Polish, Ukrainian, Russian Central); the gradient then passed through the Caucasus (Lezgin, Azeri) and South/Central Asia (Pathan, Tatar, Turkmen, Hazara, Uygur), before reaching Siberian and East Asian populations (Koryak, Altaian, Tuvan, Buryat, Yakut, Han Chinese) at the other end. The current Kazakh cohort clustered tightly together within the Central Asian segment of this gradient, overlapping closely with the independent Kazakh reference population. No individuals in the final analytic set fell outside this expected position or showed evidence of substantial admixture with populations at either extreme of the cline, consistent with the absence of residual ancestry outliers following the iterative removal described above.

### 3.3. Primary Association Analysis and LD Clumping

**Figure 3:**
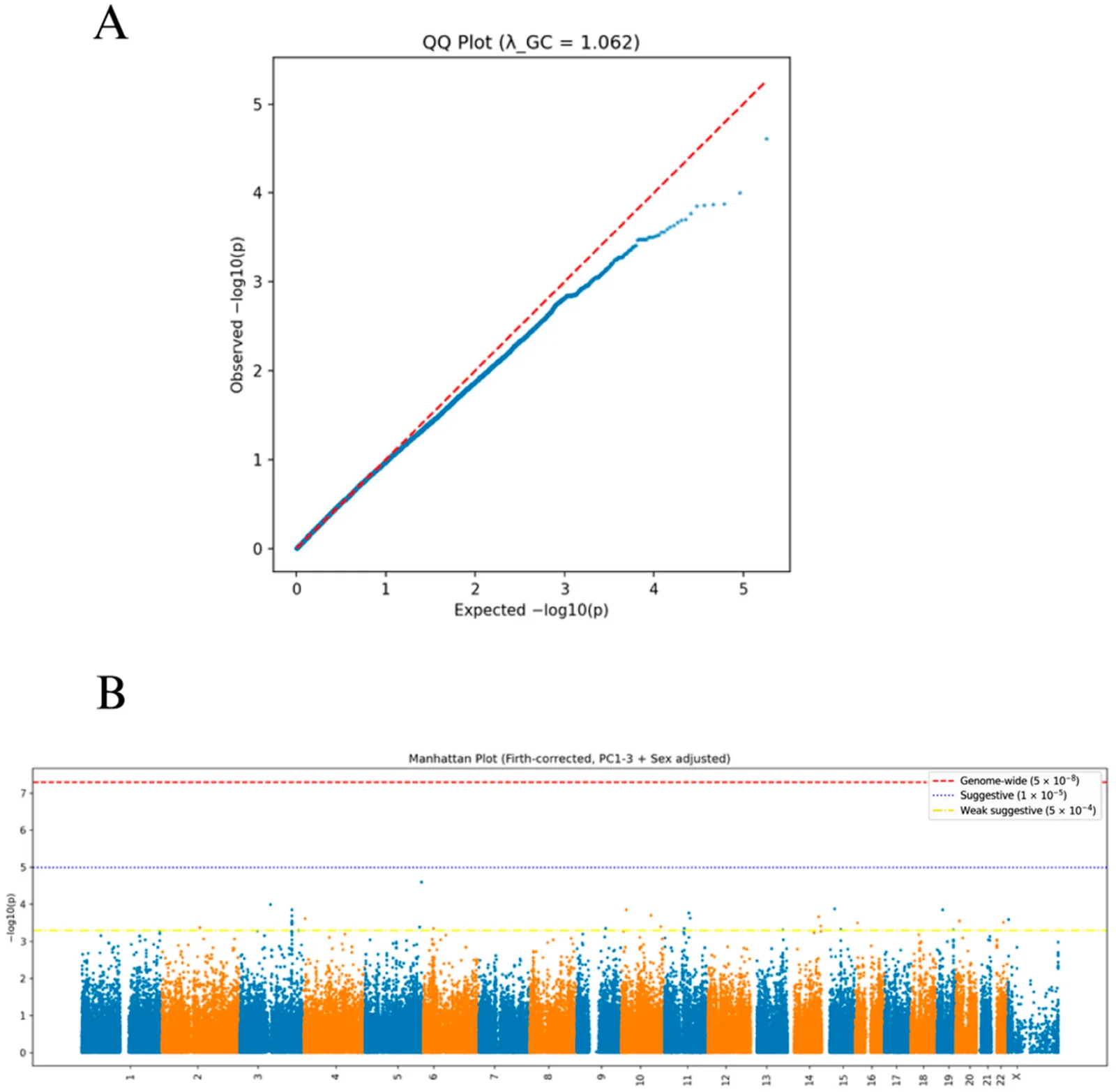
Primary association results across the targeted immune panel. (**A**) Quantile–quantile (QQ) plot of observed vs. expected −log10(p); each blue dot is one variant, and the red dashed line shows the expected null distribution (λGC = 1.062). (**B**) Manhattan plot of −log10(p) by chromosomal position; each dot is one variant, and alternating blue and orange colors distinguish adjacent chromosomes.

According to the standard for genome-wide significance (*p* < 5 × 10^−8^) and panel-corrected significance threshold (*p* < 3.06 × 10^−6^), no SNP reached the significance levels. The genomic inflation factor for the primary model was λGC = 1.062 and the QQ plot showed no substantial residual stratification (Figure 3A). The Manhattan plot (Figure 3B) displays three reference thresholds: standard genome-wide significance (*p* < 5 × 10^−8^), suggestive significance (*p* < 1 × 10^−5^), and the nominal threshold used to define suggestive loci for LD clumping (*p* < 5 × 10^−4^). The top nominal signal was observed at rs1560036 (chr5:175,833,369, within CPLX2; REF/ALT G/A; OR = 2.98, 95% CI 1.79–4.95, *p* = 2.42 × 10^−5^), which did not survive correction for multiple testing. Full association results for all variants are provided in Supplementary Table S3.

LD clumping (index *p* < 5 × 10^−4^, r^2^ ≥ 0.1, 250 kb) assigned the 39 nominally associated variants to 26 independent lead signals (Table 3). Larger numbers of correlated variants at some loci, such as 174 at the chr6 MHC locus and 72 at chr3:161 Mb, reflect the dense coverage of the immune array and extended LD in these regions; these are LD proxies of a single signal, not independent associations.

The sole chromosome-X-linked signal in this list, rs3795179 (OR = 0.30, 95% CI 0.157–0.571, *p* = 2.53 × 10^−4^), did not reach either the chromosome X-specific (8.80 × 10^−5^) or the joint (2.96 × 10^−6^) threshold.

Across all sensitivity analyses, the lead variant rs1560036 showed a consistent effect: similar odds ratios and the same direction of association regardless of which covariates were used (Table 4). In sensitivity analyses restricted to individuals with complete data (n = 162), additional adjustment for age (OR = 2.87, 95% CI 1.72–4.77, *p* = 5.06 × 10^−5^) or for age and BMI (OR = 3.28, 95% CI 1.91–5.63, *p* = 1.64 × 10^−5^) did not materially change the association at rs1560036, and 27 of the 39 nominally associated variants remained among the 39 top-ranked variants after adjustment for both. Testing for a non-additive (genotypic) effect found no evidence that the variant behaves differently from a simple additive model as the dominance-deviation term was not significant.

As a supplementary check, we performed permutation tests that do not adjust for covariates. The point-wise empirical p-value for rs1560036 was 1.1 × 10^−5^ by adaptive permutation of the allelic test, and 9.999 × 10^−5^ with 10,000 fixed permutations, which is the lowest value attainable with that number of permutations. These results indicate that the unadjusted association at this variant is not an artifact of the asymptotic approximation used in the regression; they do not directly validate the covariate-adjusted primary association. After panel-wide max(T) correction, the association was not significant (EMP2 = 0.656), which is consistent with the primary finding that no variant reached significance after correction for multiple testing.

### 3.4. Statistical Power

Post hoc sensitivity calculations of statistical power were performed at the panel-corrected simpleM threshold (α = 3.06 × 10^−6^), at standard genome-wide significance (α = 5 × 10^−8^), and, as a separate and less stringent scenario, at α = 1 × 10^−5^ (Table 5). At the panel-corrected threshold, power was below 6% for odds ratios up to 2 at all allele frequencies tested and reached at most 48.6% for OR = 3 (MAF = 0.50). Power exceeded 80% only for OR = 5 at MAF = 0.50 (94.9%) and was 67.9% for OR = 5 at MAF = 0.20. These calculations describe the effect sizes the study was able to detect; they do not imply that the null findings exclude effects of any particular size.

### 3.5. Gene-Based Association Analysis

After Bonferroni correction under either annotation window definition in the MAGMA gene-based test using the ancestry-matched Kazakh linkage-disequilibrium reference panel, no gene reached gene-wide significance. The most strongly associated genes under each window are shown in Table 6. Eight of the top nine ranked genes were shared between the 10 kb/10 kb and 35 kb/10 kb annotation windows, which reflects consistency of gene annotation rather than independent evidence of association (one ranked gene, NCBI Gene ID 105376141, corresponds to a record subsequently withdrawn by NCBI and should be treated with caution or excluded). CPLX2 (Complexin 2; chr5:175,786,539–175,894,021, 10 kb/10 kb window) contains rs1560036 (chr5:175,833,369), the lead SNP-level signal, among its six constituent SNPs; its nominal gene-based association is therefore driven largely by this single variant rather than reflecting an independent signal. CPLX2 encodes a synaptic vesicle-associated protein with a well-established role in regulated neurotransmitter release and no established role in immune function or TB pathogenesis; this convergence should therefore be interpreted as a candidate region for replication rather than as biologically self-evident and may reflect either a genuine but unannotated immune-relevant function, linkage to a nearby regulatory or coding variant in an adjacent gene, or a chance finding given the multiple comparisons involved. Full gene-based results for both windows are provided in Supplementary Table S1.

### 3.6. Functional Annotation of Suggestive Loci

Of the 39 nominally associated variants, 26 were reported as significant cis-eQTLs for at least one gene in at least one tissue in GTEx v8 (Supplementary Table S2). These are descriptive annotations rather than independent evidence of association: many of the variants are in LD within the same clumps, corresponding to only 13 of the 26 independent signals (Table 3), and individual variants are often eQTLs for several genes across multiple tissues.

Three annotations are noted as candidates for future investigation (Table 7). rs9263600 (chr6:31,103,868), the lead variant of the chr6 signal, lies in the classical MHC region and is a reported eQTL for HLA-B and HLA-C, among 105 significant gene–tissue associations. rs84460 (chr22:37,129,691) is a reported eQTL for IL2RB, which encodes a subunit of the interleukin-2 receptor. rs3740779 (chr11:76,661,008) has a prior GWAS Catalog association with Clostridioides difficile infection. None of these variants survived correction for multiple testing, and these annotations do not show that any variant influences TB risk, or that it does so through the expression of the annotated genes. Full annotation of all 39 variants (VEP consequence, gnomAD allele frequencies, GWAS Catalog cross-references, and complete GTEx eQTL results) is provided in Supplementary Table S2.

### 3.7. Polygenic Risk Score Evaluation

Polygenic risk scores derived separately from Asian- and European-ancestry base GWAS both showed odds ratios in the expected direction (OR > 1, i.e., higher PRS associated with higher odds of TB), but neither the Asia-base (OR per SD = 1.20, 95% CI 0.88–1.65, *p* = 0.26; AUC = 0.560, 95% CI 0.472–0.649) nor the Europe-base (OR per SD = 1.12, 95% CI 0.82–1.53, *p* = 0.48; AUC = 0.537, 95% CI 0.447–0.627) score showed a significant, unadjusted association with TB status in this cohort. After adjustment for ancestry principal components (PC1–PC3), neither association reached significance (Asia: *p* = 0.25; Europe: *p* = 0.58), and the AUCs of the PC-adjusted models, which include the ancestry PCs as well as the PRS, were 0.585 and 0.572 (Table 8). A DeLong’s test comparing the two PC-adjusted AUCs found no evidence of a difference between the two models (Z = 0.337, *p* = 0.736), which does not establish equivalent performance. Nagelkerke pseudo-R^2^ was low for both PC-adjusted models (Asia: 0.027; Europe: 0.021), consistent with a weak genetic signal that this sample size was underpowered to resolve with precision.

## 4. Discussion

This is the first array-based association study of pulmonary TB susceptibility in a Kazakh population; previous studies in Kazakh cohorts used candidate-gene designs [5,6,7,8,9,10]. By restricting eligibility to participants without conventional TB risk factors (Section 2.1), this study aimed to reduce heterogeneity from established epidemiological and clinical risk modifiers. A naive *p* < 0.05 threshold would flag a large number of SNPs across this 181,595-SNP panel by chance alone, so we do not treat that threshold as meaningful on its own; rather than reporting “significant” findings, we describe our top signals (those reaching *p* < 5 × 10^−4^) as nominally suggestive, since none reached significance after correction for multiple testing at any level (SNP, gene, or polygenic risk score). This null result should be interpreted in the context of the study’s limited statistical power at N = 164 (Section 3.4): the absence of a significant signal is consistent with, but does not rule out, a true genetic contribution to TB susceptibility of the modest effect sizes typically reported in larger multi-ancestry TB GWAS such as the International Tuberculosis Host Genetics Consortium meta-analysis [3], which required more than 33,000 participants to detect a genome-wide significant HLA-II signal.

Our main association analysis identified 39 SNPs reaching nominal significance, with rs1560036 as the lead signal. rs1560036 lies within CPLX2 (chr5:~175.8 Mb), which also ranked among the top MAGMA genes. Because the CPLX2 gene-based test includes rs1560036 among its six SNPs, this does not constitute independent replication. CPLX2 is involved in synaptic vesicle trafficking and has no established role in immune function or TB pathogenesis; the region should therefore be regarded as a candidate for replication.

The functional annotations of the suggestive variants (Section 3.6) are hypothesis-generating. rs9263600, the lead variant of a suggestive signal in the classical MHC region (Table 3), is a reported eQTL for HLA-B and HLA-C. The ITHGC meta-analysis reported its genome-wide significant TB signal in the HLA class II region [3], whereas our signal lies near class I genes. Although both fall within the broader MHC, they are not necessarily the same locus, gene, causal variant or mechanism, and our observation does not constitute evidence of MHC involvement in TB susceptibility in this cohort beyond an uncorrected candidate association. Similarly, the eQTL relationship between rs84460 and IL2RB is biologically plausible, given that IL-2 signaling supports the T- and NK-cell responses that produce IFN-γ, a central mediator of host defense against TB [4]; rs3740779 has been associated with a different infectious-disease trait. Neither annotation constitutes evidence of association with TB, and all three variants are candidates for replication in independent cohorts.

Previous candidate-gene studies in Kazakhstan have reported associations between TB and polymorphisms in VDR, TLR8, MARCO, IFNG and other vitamin D pathway genes [5,6,7,8,9]. The largest of these reported associations with VDR (FokI, rs2228570; BsmI, rs1544410; ApaI, rs7975232), TLR8 (rs3764880), and MARCO (rs2278589) [5]. Of these, rs1544410, rs7975232, and rs3764880 were genotyped on our array and showed no association with TB status in this cohort (OR = 1.37, *p* = 0.271; OR = 1.25, *p* = 0.322; and OR = 1.15, *p* = 0.425, respectively); rs2228570 and rs2278589 were not represented on the InfiniumImmunoArray-24v2 panel and could not be evaluated. Gene-based testing of VDR and TLR8, using broader SNP coverage than the single previously reported variant in each gene, was similarly null (VDR *p* = 0.784, 43 SNPs; TLR8 *p* = 0.303–0.415, 25–34 SNPs across the two annotation windows); MARCO had only a single SNP contributing to its gene-based test, which did not correspond to rs2278589, so this gene-based result does not directly speak to that earlier finding. This non-replication may reflect true heterogeneity (the earlier study’s VDR and MARCO associations were themselves ancestry- and sex-stratified, e.g., BsmI in Asian females and ApaI in the Caucasian subgroup of that cohort, and our cohort’s composition may differ), the modest power of the present study, or a false positive in the earlier report; distinguishing between these possibilities requires a larger, adequately powered replication cohort.

Although both PRSs showed odds ratios in the expected direction, neither was significantly associated with TB status, and the confidence intervals were wide (OR per SD 0.88–1.65 and 0.82–1.53). At this sample size, the study cannot distinguish poor transferability from insufficient statistical power, and these results should be viewed as a preliminary assessment rather than evidence for or against PRS portability to the Kazakh population. Reduced PRS portability across ancestry groups not represented in the base GWAS is well documented [11], so PRS models built primarily from European and East Asian TB genetic data may require ancestry-specific recalibration before research or clinical application in this population. Formal evaluation will require larger cohorts and, ideally, the inclusion of Central Asian populations in the base GWAS from which TB PRSs are derived.

This study has several limitations. First, the InfiniumImmunoArray-24v2 is a targeted, immune-pathway-focused panel rather than a genome-wide array; SNP coverage is therefore non-uniform, and null results away from immune-relevant regions may reflect sparse coverage rather than a true absence of association. Non-uniform coverage also reduced overlap with base-GWAS variants for PRS construction, and PRSs were constructed at a single p-value threshold. Second, chromosome X was analyzed separately, with a Bonferroni threshold defined after the autosomal analysis; the sole chromosome X signal (rs3795179) did not reach either this threshold or a joint autosomal-plus-X threshold, and it lies within the pseudo-autosomal region PAR1 but was analyzed under X-chromosome coding, so it should be interpreted with particular caution. Third, the sample size is small relative to contemporary multi-ancestry GWAS, limiting power (Section 3.4), and no independent replication cohort was available; all reported associations should be regarded as hypothesis-generating pending replication in an independent Kazakh or Central Asian cohort. Fourth, restricting eligibility to individuals without conventional risk factors reduces heterogeneity but does not eliminate confounding by unmeasured factors. It also limits generalizability to the wider TB population in Kazakhstan, in which such risk factors are common, as does the exclusion of individuals with registered TB contact, given the role of contact tracing in case detection; this exclusion may also increase the proportion of controls never exposed to M. tuberculosis. Restriction could also introduce selection bias if the probability of meeting the eligibility criteria differed between cases and controls or were associated with genetic ancestry. The use of identical criteria for both groups, individual matching, and the absence of residual population stratification after ancestry adjustment (λGC = 1.062) mitigate, but do not exclude, these possibilities. Fifth, the case group included both bacteriologically confirmed and clinically diagnosed cases, as well as new and recurrent episodes, and controls were not tested for latent TB infection. The study therefore compares active pulmonary TB with the absence of clinical or radiological disease, rather than infected with uninfected individuals. Such misclassification would be expected mainly to bias associations toward the null and reduce power, rather than to produce spurious associations. Finally, although controls were individually matched to cases, the analysis was unconditional and did not adjust for all matching variables. Adjustment for age, and for age and BMI, in sensitivity analyses did not materially change the lead association (Section 3.3), but region of residence was not included as a covariate, so residual confounding by residence cannot be excluded.

Despite these limitations, this study contributes genomic data from an ancestry group absent from the multi-ancestry consortia used to build current TB PRSs, supporting future inclusion of Kazakh and Central Asian populations in multi-ancestry TB genetics consortia and meta-analyses.

## 5. Conclusions

In this case–control immunogenetic array study of pulmonary TB susceptibility in a Kazakh population that also excluded participants with conventional TB risk factors, several SNPs, including rs1560036, reached nominal significance, though none survived correction for multiple testing. Although this study had limited power to detect the modest effect sizes typical of complex infectious-disease traits, it generated candidate signals for replication in larger cohorts. Published Asian- and European-ancestry PRSs showed effects in the expected direction but no statistically significant evidence of predictive performance in this sample. Importantly, this study provides the first array-based immunogenetic association data on TB for a Kazakh population, extending earlier candidate-gene studies [5,6,7,8,9,10]. The underlying dataset with genotypes, summary statistics, and functional annotations is being made openly available to support replication and inclusion of Central Asian populations in future, larger-scale TB genetics research.

## Figures and Tables

**Table 1:** Demographic and anthropometric characteristics of the study participants (n = 164).

Characteristic	All (n = 164)	Cases (n = 84)	Controls (n = 80)
Age, years, mean ± SD	31.7 ± 11.4	30.6 ± 11.4	32.8 ± 11.3
Height, cm, mean ± SD	168.2 ± 8.8	168.4 ± 8.6	167.9 ± 9.1
Weight, kg, mean ± SD	61.5 ± 10.8	59.6 ± 11.1	63.5 ± 10.1
BMI, kg/m^2^, mean ± SD	21.6 ± 3.5	21.0 ± 3.9	22.3 ± 2.8
Sex, n (%)			
Male	92 (56.1)	47 (56.0)	45 (56.3)
Female	72 (43.9)	37 (44.0)	35 (43.8)
Residence, n (%)			
Urban	81 (49.4)	44 (52.4)	37 (46.3)
Rural	81 (49.4)	40 (47.6)	41 (51.3)
Unknown	2 (1.2)	0 (0.0)	2 (2.5)

Sex for two participants with missing records was assigned from genetically inferred sex.

**Table 2:** Sample and variant flow through quality control.

Processing Stage	Samples	SNPs
Raw genotype calls	192	247,814
Split multiallelics + dbSNP annotation	192	248,349
After rsID/duplicate-ID resolution	192	248,162
After ambiguous-strand (A/T, C/G) removal	192	220,595
Sample call-rate filtering (geno/mind)	185	217,647
Heterozygosity-outlier removal	184	217,647
KING relatedness pruning (2nd-degree cutoff)	179	217,647
Hardy–Weinberg equilibrium filtering (controls)	179	217,646
Minor allele frequency ≥1%	179	181,595
Ancestry-outlier removal, round 1	173	181,595
Ancestry-outlier removal, round 2	168	181,595
Ancestry-outlier removal, round 3 (final analytic set)	164	181,595

**Table 3:** Twenty-six independent signals to which the 39 nominally associated variants (*p* < 5 × 10^−4^) were assigned by LD clumping, ranked by *p*-value. “SNPs in LD Clump” counts the lead SNP and all array variants in LD with it (r^2^ ≥ 0.1, 250 kb), regardless of *p*-value; these are LD proxies, not additional associations.

CHR	Lead SNP	BP	*p*	SNPs in LD Clump
5	rs1560036	175,833,369	2.42 × 10^−5^	5
3	rs13081814	95,829,911	9.95 × 10^−5^	2
15	rs4923807	38,901,041	1.32 × 10^−4^	1
10	rs11818063	15,029,619	1.36 × 10^−4^	3
19	rs62121092	18,052,445	1.38 × 10^−4^	14
3	rs6441370	161,275,966	1.39 × 10^−4^	72
11	rs3740779	76,661,008	1.71 × 10^−4^	5
10	rs835259	93,022,978	1.98 × 10^−4^	16
14	rs1885152	95,858,869	2.14 × 10^−4^	2
11	rs1265425	80,251,941	2.33 × 10^−4^	3
4	rs4330304	4,367,673	2.39 × 10^−4^	2
X	rs3795179	2,623,614	2.53 × 10^−4^	6
20	rs6134366	11,812,314	2.75 × 10^−4^	2
22	rs84460	37,129,691	3.00 × 10^−4^	13
16	rs12597409	8,402,882	3.10 × 10^−4^	4
14	rs10873531	102,101,959	3.88 × 10^−4^	10
10	rs11200411	122,120,697	3.94 × 10^−4^	7
5	rs17072023	170,054,447	4.06 × 10^−4^	3
2	rs512681	118,692,587	4.15 × 10^−4^	3
9	rs7848364	89,661,201	4.36 × 10^−4^	1
11	rs2905517	61,000,572	4.36 × 10^−4^	61
6	rs9263600	31,103,868	4.42 × 10^−4^	174
15	rs11071311	57,316,925	4.63 × 10^−4^	12
13	rs12586091	99,399,080	4.83 × 10^−4^	39
19	rs7259990	51,532,449	4.83 × 10^−4^	59
3	rs9853234	182,217,390	4.94 × 10^−4^	2

**Table 4:** Sensitivity analysis of the lead variant, rs1560036, across the primary model and alternative model specifications.

Model	OR	95% CI	*p*-Value
Primary (Firth-fallback, sex + PC1-3)	2.98	1.79–4.95	2.42 × 10^−5^
Sex-unadjusted (PC1-3 only)	2.98	1.80–4.95	2.42 × 10^−5^
Full Firth (non-fallback)	2.84	1.73–4.64	3.25 × 10^−5^
Genotypic model—additive term	3.00	1.71–5.27	1.28 × 10^−4^
Genotypic model—dominance-deviation term	0.98	0.47–2.03	0.948
Permutation, allelic test, point-wise (adaptive, up to 10^6^)	—	—	1.1 × 10^−5^
Permutation, best-fit test, point-wise (10,000 max(T))	—	—	9.999 × 10^−5^ *
Permutation, panel-wide corrected (EMP2)	—	—	0.656
+age (n = 162)	2.87	1.72–4.77	5.06 × 10^−5^
+age + BMI (n = 162)	3.28	1.91–5.63	1.64 × 10^−5^

* Lowest attainable value with 10,000 permutations.

**Table 5:** Post hoc power (%) to detect an additive association at N = 164 (84 cases, 80 controls), by odds ratio (OR) and minor allele frequency (MAF).

OR	MAF	Power at α = 3.06 × 10^−6^ (%) (Panel-Corrected)	Power at α = 1 × 10^−5^ (%)	Power at α = 5 × 10^−8^ (%)
1.5	0.05	0.005	0.014	0.00016
1.5	0.10	0.017	0.043	0.00064
1.5	0.20	0.065	0.151	0.0032
1.5	0.50	0.219	0.465	0.0139
2	0.05	0.041	0.099	0.0018
2	0.10	0.222	0.473	0.0142
2	0.20	1.27	2.36	0.127
2	0.50	5.26	8.53	0.809
3	0.05	0.395	0.805	0.029
3	0.10	2.94	5.04	0.374
3	0.20	17.0	24.0	4.10
3	0.50	48.6	58.4	20.6
5	0.05	2.76	4.77	0.345
5	0.10	19.8	27.5	5.12
5	0.20	67.9	76.3	37.5
5	0.50	94.9	97.1	80.4

**Table 6:** Top MAGMA gene-based association results shared between the 10 kb/10 kb and 35 kb/10 kb annotation windows, ranked by 10 kb/10 kb *p*-value.

Gene Symbol (Entrez ID)	CHR	Position (bp)	NSNPS	*p* (10 kb/10 kb)	*p* (35 kb/10 kb)
DOCK1 (1793)	10	126,895,409–127,462,517	24	5.09 × 10^−4^	5.84 × 10^−4^
GPC6 (10082)	13	93,216,825–94,418,020	27	5.14 × 10^−4^	6.10 × 10^−4^
CPLX2 (10814)	5	175,786,539–175,894,021	6	6.49 × 10^−4^	9.00 × 10^−4^
DCBLD2 (131566)	3	98,785,970–98,911,689	4	7.16 × 10^−4^	7.16 × 10^−4^
CEP89 (84902)	19	32,868,998–32,982,029	1	9.89 × 10^−4^	9.89 × 10^−4^
KDM2A (22992)	11	67,109,268–67,268,082	1	1.06 × 10^−3^	1.06 × 10^−3^
ZNRF4 (148066)	19	5,445,415–5,466,856	4	1.47 × 10^−3^	1.47 × 10^−3^
FBN2 (2201)	5	128,247,909–128,548,042	7	2.37 × 10^−3^	2.37 × 10^−3^
LOC105376141 (105376141) †	9	89,644,416–89,672,115	1	4.36 × 10^−4^	2.33 × 10^−3^

† LOC105376141 corresponds to an NCBI Gene record that has since been withdrawn; it is listed last, out of *p*-value order, and should be interpreted with caution.

**Table 7:** Notable functional annotation results among the 39 nominally associated SNPs.

SNP	CHR:Position	eQTL Target Gene(s)	Notable Context
rs9263600	6:31,103,868	HLA-B; HLA-C (+103 other MHC-region genes/tissues)	Classical MHC region (class I); ITHGC TB signal reported in the class II region [3]
rs84460	22:37,129,691	IL2RB	Interleukin-2 receptor subunit; established role in T-cell immune regulation
rs3740779	11:76,661,008	LRRC32 (+2 others)	Prior GWAS Catalog association with Clostridioides difficile infection susceptibility (*p* = 6 × 10^−6^)

**Table 8:** Polygenic risk score evaluation: AUC and logistic regression results for both base-GWAS populations.

Base GWAS	AUC (95% CI)	OR per SD (95% CI)	*p*-Value (Alone/+PC1-3)
Asia	0.560 (0.472–0.649)	1.20 (0.88–1.65)	0.260/0.249
Europe	0.537 (0.447–0.627)	1.12 (0.82–1.53)	0.482/0.580

## Data Availability

The genotype data presented in this study are openly available in the European Variation Archive (EVA) at EMBL-EBI under Project accession PRJEB126695 (analysis KAZ_GWAS_TB, accession ERZ29899492). This open-access arrangement is consistent with what was agreed to in the informed consent form. Full single-variant association results (Table S3), PRS variants and weights (Table S4), MAGMA gene-based results (Table S1) and functional annotations (Table S2) are provided as Supplementary Materials. The Kazakh whole-genome-sequencing reference panel used for LD estimation in the gene-based analysis is part of an ongoing, unpublished study and is not publicly available.

## Supplementary Materials

The following supporting information can be downloaded at https://www.mdpi.com/article/10.3390/biomedicines14102387/s1: Table S1: Full MAGMA gene-based results for both annotation windows; Table S2: Full VEP, gnomAD, GWAS Catalog, and GTEx functional annotation results for all 39 nominally associated SNPs; Table S3: Full single-variant association results from the primary model (additive term); Table S4: Variants, effect alleles and weights used to construct the Asia-base (S4a) and Europe-base (S4b) polygenic risk scores.

## Abbreviations

The following abbreviations are used in this manuscript:

TB	Tuberculosis
GWAS	Genome-wide association study
SNP	Single-nucleotide polymorphism
PCA	Principal component analysis
PC	Principal component
MAF	Minor allele frequency
LD	Linkage disequilibrium
OR	Odds ratio
CI	Confidence interval
PRS	Polygenic risk score
AUC	Area under the receiver operating characteristic curve
MAGMA	Multi-marker Analysis of GenoMic Annotation
KING	Kinship-based INference for Genome-wide association studies
BMI	Body mass index
UNEHS	Unified National Electronic Healthcare System
EVA	European Variation Archive
